# The Importance of Ultrasonography in the Evaluation of Mammary Tumors in Bitches

**DOI:** 10.3390/ani13111742

**Published:** 2023-05-24

**Authors:** Marcus Antônio Rossi Feliciano, Brenda dos Santos Pompeu de Miranda, Luiz Paulo Nogueira Aires, Bruna Bressianini Lima, Ana Paula Luiz de Oliveira, Giovanna Serpa Maciel Feliciano, Ricardo Andrés Ramirez Uscategui

**Affiliations:** 1 Laboratory of Veterinary Imaginology, Faculty of Animal Science and Food Engineering (FZEA), Sao Paulo University (USP), Pirassununga 13635-900, Sao Paulo, Brazil; 2 Department of Veterinary Clinic and Surgery, School of Agricultural and Veterinarian Sciences, Sao Paulo State University “Júlio de Mesquita Filho” (FCAV/UNESP), Jaboticabal 14884-900, Sao Paulo, Brazil; 3 Research Group INCA-CES, Faculty of Veterinary Medicine and Animal Science, Universidad CES, Medellín 050021, Colombia

**Keywords:** diagnostic imaging, canine, neoplasms, elasticity imaging techniques, angiogenesis, perfusion

## Abstract

**Simple Summary:**

Mammary tumors are one of the most common neoplasms in bitches and represent an important concern in small animal practice. Clinical staging requires an extensive clinical evaluation and definitive diagnosis is obtained by means of histopathological evaluation. Ultrasonography is a well-established imaging technique in veterinary medicine that allows detailed evaluation of the genital system. Other advanced sonographic techniques, such as Doppler, elastography and contrast-enhanced ultrasound (CEUS) have raised interest in the scientific community and researchers have been performing studies to evaluate whether these techniques can accurately differentiate tumoral lesions. This paper reviews current information regarding the sonographic findings of mammary tumors in each different ultrasound technique. Overall, based on the information present to this date, elastography is the most reliable ultrasound technique to distinguish benign lesions from malignant tumors. Nevertheless, each technique demonstrates important findings that could guide the therapeutic approach and can be applied in veterinary practice. Additionally, further studies are required to evaluate the potential of each technique to monitor therapeutic progression.

**Abstract:**

The high incidence of mammary tumors in small animals is concerning. Patient history, clinical examination, physical evaluation, and imaging studies are important for clinical staging. Ultrasonography is commonly applied to investigate the presence of abdominal metastasis. However, it has been shown to provide important information regarding mammary tumors’ architecture and advanced sonographic techniques can provide information regarding neovascularization, stiffness, and perfusion. Different techniques have been investigated to determine accuracy to predict the lesions’ histological classification. This paper reviews the information regarding each sonographic technique in the evaluation of mammary tumors, describing the most common findings and their potential to accurately assess and predict malignancy. Even though the gold standard for the diagnosis of mammary lesions is the histopathological examination, some ultrasonographic features described can predict the potential of a lesion being malignant. Among the different sonographic techniques, elastography can be considered the most reliable modality to accurately differentiate benign from malignant tumors when malignant lesions present increased stiffness. However, the combination of all sonographic techniques can provide important information that can lead to a better therapeutic approach and clinical staging. Furthermore, the potential of the sonographic study, especially CEUS to monitor therapeutic progression, demonstrate the need of further studies.

## 1. Introduction

Mammary tumors represent the most diagnosed neoplasms in bitches, with a prevalence three times higher than in women and most of these neoplasms present malignant characteristics [1,2]. Malignant tumors are worrisome due to the high chance of metastasis development. Its occurrence in bitches with mammary tumors can reach up to 50%, representing the main mortality factor for the affected patients [3,4]. Therefore, early diagnosis is important, as it allows adequate staging, which leads to better therapeutic management and prognosis.

Ultrasonography is an important imaging technique in veterinary practice and is considered the modality of choice to investigate diseases of the reproductive tract, monitor the morphological and physiological changes due to the cyclic activity of the female, as well as to diagnose pregnancy, evaluate the development of embryos/fetuses, and detect some gestational abnormalities [5,6,7]. In women, breast ultrasonography is an important tool for the diagnosis of mammary lesions and is the imaging modality of choice, as it does not emit ionizing radiation and can be performed in pregnant and lactating patients. In addition, it presents better sensitivity and specificity than mammography [8,9].

In small animal practice, the ultrasound evaluation of bitches with mammary tumors is mainly performed for oncological staging to investigate possible metastatic lesions in abdominal organs [10]. However, its applicability extends further than just monitoring metastatic lesions, as it can also aid in the evaluation of mammary lesions [11]. Among the different sonographic techniques, advanced modalities such as elastography and contrast-enhanced ultrasound (CEUS) have been gaining attention and visibility in both human and veterinary medicine due to the promising data that these techniques provide. Elastography is a technique that evaluates tissue stiffness, providing both qualitative and quantitative information of the area of interest [12]. CEUS is a technique that uses a contrast medium containing a stabilized gas located inside a shell. The gas is composed of an inert material (microbubbles) similar to the size of erythrocytes that can penetrate capillaries and intensify acoustic response and Doppler signal, providing both qualitative and quantitative information regarding tissue perfusion in real time [13,14,15].

Several studies have been carried out to evaluate the applicability of the ultrasonographic examination of mammary lesions in bitches. Among the different sonographic techniques, elastography and contrast-enhanced ultrasound have shown promising results regarding the diagnostic accuracy of the lesion characteristics. Early diagnosis can provide adequate therapeutic management and a better prognosis. Thus, the aim of this review is to highlight the benefits of the sonographic techniques and explore their application in small animal practice.

## 2. Diagnosis

According to the Consensus Regarding the Diagnosis, Prognosis and Treatment of Canine and Feline Mammary Tumors in 2019 by Cassali et al., diagnosis of mammary tumors requires an extensive clinical evaluation with a complete history and physical examination for adequate staging [16]. After this first approach, a fine needle aspiration (FNA) can be performed to differentiate tumor types, inflammation, and hyperplasia, and to rule out cutaneous tumors developed at the mammary gland region. However, this procedure is considered a preliminary assessment due to the fact that it does not allow a definitive and conclusive diagnosis of mammary tumors and has a tendency to underdiagnose malignant tumors [16,17,18,19].

Despite being an invasive and somewhat laborious procedure when compared to FNA, histopathological evaluation is considered the gold standard for definitive diagnosis of mammary tumors and is crucial for all cases [16,19]. Usually, this procedure is performed after surgery and it does not evaluate just the primary tumor, but all the mammary glands and regional lymph nodes, as well as the transition from the gross tumor lesion area to the supposedly adjacent healthy tissue to evaluate surgical margins and establish the best therapeutic management from this information [16].

Other complementary diagnostic methods can be implemented in the oncological routine in bitches, such as immunohistochemistry, which contributes to surgical and therapeutic management by means of proliferation markers, and liquid biopsy, which has provided excellent means of detecting new genetic mutations in the initial stages and in the follow-up of patients with mammary cancer on an experimental level using next-generation sequencing assay [20,21]. Additionally, several biomarkers can aid in the early diagnosis of mammary lesions, such as cell cycle markers, proliferation markers, apoptosis markers, metastatic potential and prognosis, hormonal, and inflammatory receptors, among others [22].

## 3. Ultrasonography

### 3.1. B-Mode

**Figure 1:**
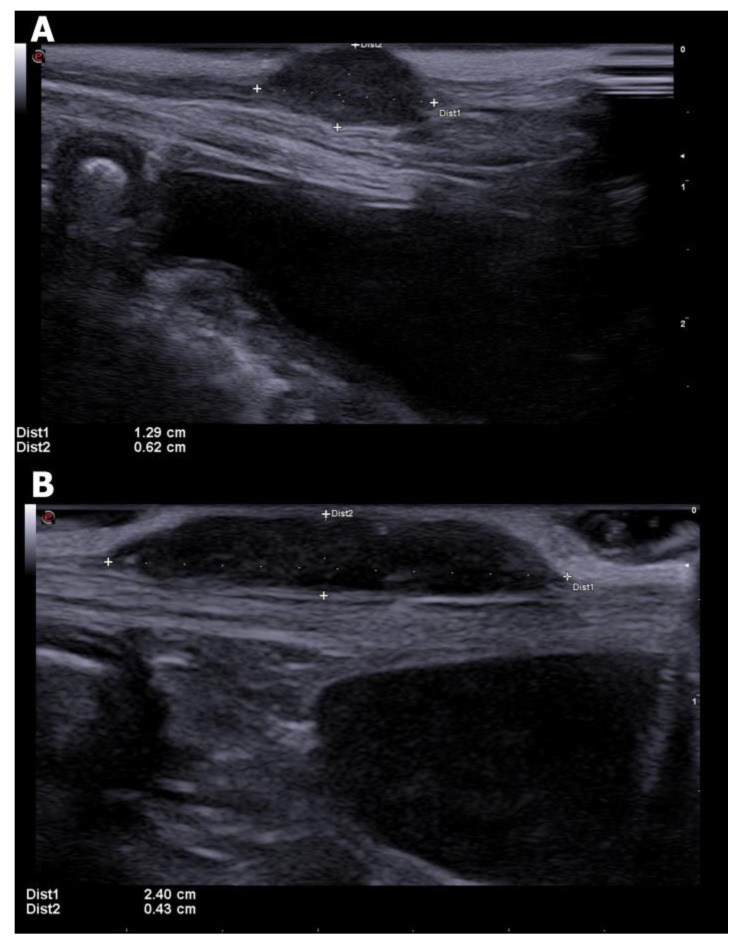
B-mode ultrasonography of mammary tumors in bitches. (**A**) a small oval hypoechoic mammary nodule is seen between electronic calipers, presenting circumscribed margins. (**B**) a small elongated hypoechoic mammary mass is seen between electronic calipers, with slightly irregular margins. (Frequency: 14 MHz; Depth: 2.8 cm).

Conventional or B-mode ultrasonography evaluates tissue architecture in a gray-scale processing of the received echoes and is the most accessible method for non-invasive evaluation of the mammary glands, which allows assessment of the lesion characteristics [23]. Considering that the mammary glands are located in a superficial region, the use of high-frequency linear transducers (that is, the highest frequency available that allows complete evaluation of the structure), providing adequate visualization of small structures with great resolution [23,24]. An exemplification of possible appearances of mammary tumors can be found in Figure 1.

For the evaluation of mammary tumors on conventional ultrasonography, several parameters have been assessed by different authors. Sonographic features evaluated are: size, width to length ratio (W/L); shape; margins; limit, nodule/mass echogenicity compared to the surrounding tissues; echotexture; presence or absence of hyperechoic halo; presence or absence of distal acoustic enhancement or shadowing; and invasiveness [23,24,25,26,27,28,29,30,31,32,33,34,35].

Given the different parameters that can be evaluated in conventional ultrasonography, it is important to point out that there are divergences between determination of the effectiveness of these parameters as indicators in the differentiation of mammary neoplasms according to different authors, as well as different terminology proposed. In 1998, Gonzales de Bulnes et al. evaluated 19 bitches with mammary tumors and reported that malignant tumors tend to have irregular margins, polymorphic shape (irregular), and invasive character in relation to adjacent tissues, stating that these criteria could help in the discrimination of benign tumors [23].

In 2006, Nyman et al. [25] studied 49 mammary tumors and reported that on B mode ultrasonography, benign mammary tumors were isoechoic to hypoechoic when compared to the adjacent tissue. As for malignant lesions, a wide variability of characteristics such as echogenicity and presence or absence of invasion of the surrounding tissues was observed, which was confirmed histologically, despite not showing a statistically significant result. In 2009, Baştan et al. [26] reported that the most predictive features of malignant tumors were regarding their shape and margins, where 76% of masses that presented spiculated or microlobulated margins were malignant and 78% of masses that presented irregular shape were malignant.

Similar results were reported by a study performed by Guedes et al. in 2020, in which the authors demonstrated that mammary masses with heterogeneous echotexture and mixed components characterized by the existence of cystic and solid areas in the same structure were associated with malignancy (*p* < 0.05) [27], corroborating a study from 2016 by Soler et al., which reported that, among the numerous parameters evaluated in B-mode, the heterogeneous echotexture present in malignant tumors was significant (*p* < 0.01) [28]. In their study, although the presence of acoustic shadowing due to the presence of calcification was not statistically significant, the authors reported that the incidence of this finding was lower in malignant tumors. Data gathered in a study conducted by Hillaert et al. [29] indicate that B-mode sonographic characteristics capable of discriminating between malignant and benign lesions are border definition, echogenicity, and echotexture, where features such as ill-defined structures and mixed echogenic heterogenous nodules tend to be malignant.

Conversely, in studies conducted by the author’s research group, different results were found, where none of the B mode qualitative features were able to differentiate benign from malignant lesions, probably due to the use of a high-definition transducer (12 MHz), which allowed better resolution and visualization of the tumoral architecture [24,29,30]. However, the same studies report that measurements obtained in B mode evaluation (mass length and height in longitudinal view; width, height and width/height ratio in transverse view) are significantly higher in malignant tumors. Parenchymal alterations of malignant tumors (edema, necrosis, calcification, and hemorrhage) are the causes of the increase in masses and variety in echogenicity observed in the B-mode examination [24].

Evaluating 300 mammary neoplasms, Feliciano et al. found that B-mode ultrasonography has a sensitivity of 67.9%, specificity of 67.6% and area under the curve (AUC) of 69.5% for detecting malignancy in mammary tumors [24]. According to the authors, the low effectiveness of B-mode in differentiating mammary tumors is justified by the histopathological variability of each tumor type, its morphophysiological characteristics, structural heterogeneity in benign and malignant tumors, in addition to the biological and nonspecific aspects of each type of neoplasm, making it difficult to consider only B-mode ultrasonographic parameters in tumor differentiation and prediction of malignancy.

Comparing the types and degrees of mammary carcinomas in female dogs in a study carried out in 2018, Feliciano et al. demonstrated that the width:length ratio of malignant neoplasms was significantly (*p* < 0.05) different between carcinoma types or grades, with higher values for special type carcinomas, resulting in an identification cohort value greater than 0.53, with 80% sensitivity, 76% specificity, 78% accuracy, and 80% area under the curve [32]. Additionally, the authors report that other B-mode parameters did not demonstrate applicability in differentiating types and grades of mammary carcinomas in bitches.

When investigating small mammary nodules (<2 cm), a study conducted by Vannozi et al. [33] described that none of the commonly evaluated features of shape, limit, echogenicity, echotexture, presence of distal acoustic enhancement, or shadowing were able to discriminate between benign and malignant lesions. Another feature observed by the authors was changes in the tissues surrounding the mammary nodules, a characteristic that was considered important and that may indicate malignancy. However, the authors claim that its subjectivity may limit its application in small animal practice.

An important issue to consider is that different terminologies have been proposed by different authors regarding sonographic features (i.e., irregular shape vs. polymorphic shape; ill-defined margins vs. invasiveness or lesion limit). Therefore, a standardized system is preferred to describe these findings. In 2011, a study conducted by Mohammed et al. [34] proposed a standardized method for canine mammary ultrasound evaluation regarding lexicon, reporting and grading based on the well-known BI-RADS™ (Breast Imaging Reporting and Data System) method established in human medicine by the American College of Radiology [36]. This systematic approach was further explored in 2018 by Oliveira and colleagues [37]. The terminology proposed by these studies for sonographic reporting can be found in Table 1.

Quantitative assessment of mammary tumors has been attempted to investigate whether it is possible to discriminate between malignant and benign lesions based on quantitative information of echogenicity and echotexture [37,38]. These studies have reported that computer-assisted echotexture analysis is an important feature that presents predictive value to differentiate benign from malignant lesions, where heterogenicity is often observed in malignant tumors. However, these studies were conducted with the use of lower frequencies than usually applied to study superficial structures (7.5 to 8.0 MHz), which could limit the applicability of these findings when using higher frequency transducers. Further studies could be implemented in order to evaluate the possibility of computer-assisted echostructure analysis using higher frequency transducers.

These sonographic findings can be used to classify the mammary lesion in six different categories according to the modified BI-RADS classification proposed for bitches with mammary tumors [34,36,37]. The BI-RADS categories and their common findings can be found in Table 2.

These studies conducted to validate the Modified BI-RADS classification demonstrated that Categories 3 to 5 have higher chances of presenting malignancy, where Category 5 presents higher malignancy incidence [34,36,37]. However, it is prudent to consider that these findings and reporting systematization do not provide definitive diagnosis and that biopsy would be required for diagnostic elucidation.

### 3.2. Doppler Ultrasonography

Considering the different growth factors that induce carcinogenesis, intratumoral and peritumoral angiogenesis are widely studied with the purpose of understanding and intervening in the mechanisms of tumoral maintenance, growth, invasion, and subsequent metastasis [39]. Given the need to evaluate and understand tumoral tissue vascularization, Doppler ultrasonography provides real-time information on vascularization and hemodynamic aspects of the detectable blood flow, which allows measurement of the vascular velocity and resistivity; hence, Doppler technique is a relevant tool in the diagnostic approach of mammary neoplasms [40].

Color Doppler allows assessment of the presence or absence of blood flow in a specific region of interest selected by the operator, and its direction in different tissues. Its applicability in mammary neoplasms in bitches has been studied and has shown interesting results as to the differentiation between benign and malignant lesions [24,25,28,32,40,41]. Regarding blood flow distribution, some studies report that peripheral vascular patterns were more evident in benign tumors, while the mixed pattern, that is, presence of vascularization in a somewhat random distribution centrally and peripherally, was predominant in malignant tumors (*p* < 0.05) [28,30]. Conversely, a study conducted in 2006 by Nyman et al., detected a mixed vascular pattern in all benign lesions and in only 28 out of the 38 atypical benign and malignant tumors [25]. Additionally, a positive correlation (*p* < 0.001) was found between large tumors that presented a smaller vascular supply than smaller tumors and an overall correlation (*p* = 0.015) between the number of vessels detected sonographically and the number detected histologically.

Although some studies indicate that there is no significant difference between the distribution of blood flow between benign and malignant mammary lesions [25,31,34,38,40] or the presence or absence of vascularization [31,40], one study reports that malignant tumors have a higher proportion of vascularization (*p* < 0.01) with sensitivity of 86.0%, specificity of 47.9% and accuracy of 81.5% in predicting malignancy [24]. Therefore, this technique is useful to detect intratumoral and peripheral angiogenesis and bring additional information that can help in oncological targeting, when associated with other sonographic and clinical characteristics.

**Figure 2:**
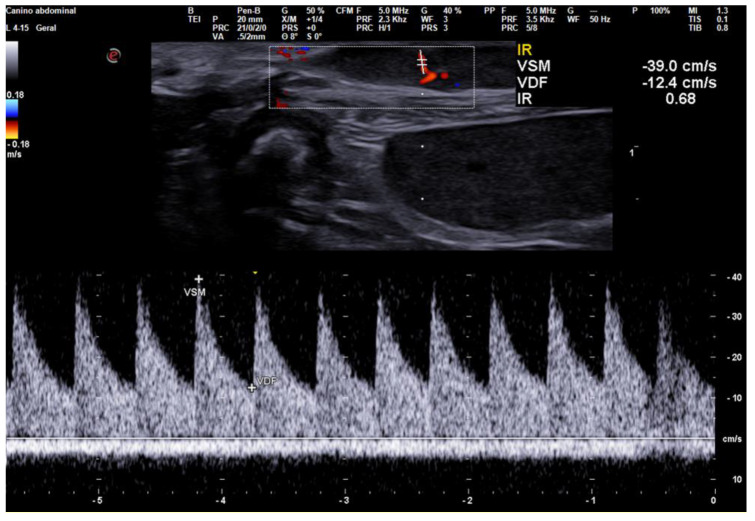
Ultrasound image of a malignant mammary tumor in a bitch, using triplex Doppler assessment (B-mode, color-coded doppler, and pulsed-wave doppler -triplex; increased velocities -systolic peak velocity (VSM); and end-diastolic velocity (VDF)-indicate malignancy. Sample volume = 0.5/2 mm.

Pulsed-wave Doppler, also known as spectral Doppler allows measurement of the velocity within a vessel within a specific sample volume selected by the sonographer and is useful for differentiating malignant from benign tumors using vascular indices (known as Doppler fluxometry evaluation), although the limitation of this method is the impossibility of detecting arterial blood flow in some types of tumors. According to a study conducted by Feliciano et al. in 2012, the maximum vascular velocity in malignant tumors tends to be higher (28.71 cm/s), in contrast to benign mammary tumors that present lower velocity (19.91 cm/s), in addition to having a positive correlation between vascular endothelial growth factor (VEGF) and the presence of tumor vascularization and maximum velocity (Figure 2) [40]. These authors state that the increased permeability, vessel dilation, bending to specific tyrosinase receptors of endothelial cells, stimulation of the growth of neoplastic cells and inhibition of apoptosis are alterations that could possibly be responsible for the correlation found between VEGF and tumor blood flow velocity [40].

Despite these findings regarding the maximum velocity, no difference in the measurements of minimum velocity, resistivity index, and pulsatility index were found in this study, similar to the study by Soler et al. [28]. A positive correlation between increased systolic velocity and diastolic velocity in malignant tumors was found in one study (*p* < 0.01) with 79.2% sensitivity, 70.8% specificity, and 71.0% accuracy in predicting malignancy [24].

Although the results presented by the Doppler mode in detecting malignancy in mammary tumors are relevant, it is prudent to consider the technical factors associated with Doppler studies, such as the angle of insonation, which highly influences the values obtained. Therefore, it is important to interpret these findings carefully. Exemplifying one of these considerations, Feliciano et al. verified that in approximately 44% of malignant tumors and 54% of the benign ones, it was not possible to detect vascularization using the Doppler technique, which makes it impossible to evaluate quantitative vascular parameters [40]. The inability to detect vascularization is an important limitation of this diagnostic technique in the differentiation of benign and malignant mammary tumors in bitches.

In a study that aimed to compare the different types and grades of mammary carcinomas in bitches, all tumors evaluated showed neovascularization and high values of vascular indices. However, color Doppler and Doppler fluxometry parameters did not provide information that indicate the applicability of the method in differentiating types and grades of carcinomas [32].

### 3.3. Elastography

The idea that tissue stiffness can change in diseased and tumoral tissues is what led several researchers to investigate whether it is possible to differentiate benign and malignant lesions based on tissue stiffness changes detected by elastography. However, it is important to understand that several elastography software programs are available in different equipment and some principles can vary from one technique to the other [24].

In-depth explanation of each elastography method is beyond the scope of this review; hence, a basic overview of the technique will be presented. Overall, two main elastography methods are available in the market: the quasi-static method (in which a manual compression is performed to obtain a qualitative operator-dependent evaluation of tissue elasticity) and the dynamic method (where a time-varying force is applied to the area of interest by either a short mechanical force or an oscillatory force, propagating shear waves that can be quantified and that are operator-independent) [12].

Both methods have been applied in small animal practice to study the stiffness of mammary neoplasms and promising results were obtained. A study conducted in 2022 by Massimini et al. performed strain elastography evaluation (STE; a quasi-static method that provides qualitative information regarding the relative tissue stiffness) and shear-wave elastography evaluation (SWE; a dynamic method that provides quantitative information regarding tissue elasticity) of mammary tumors in bitches found that SWE values are positively correlated to lesion fibrosis, but no difference was obtained between benign and malignant lesions [42]. Furthermore, the authors report that the repeatability of SWE was high and for STE it was moderate, demonstrating that quantitative methods are more assertive for the evaluation of mammary lesions.

**Figure 3:**
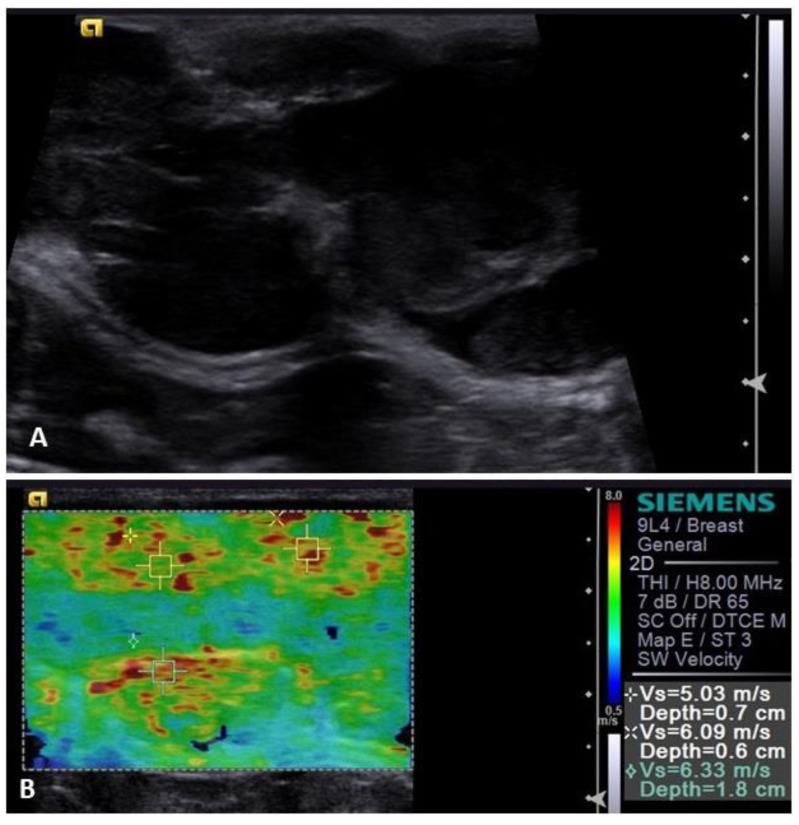
Ultrasound image of a malignant mammary tumor in a bitch. (**A**) B-mode image, demonstrating heterogeneous echotexture and mixed echogenicity, with solid and liquid components; (**B**) image of the ARFI elastography assessment of the same patient, obtaining the elastogram and shear velocity of the mass—note the presence of increased stiffness both in the elastogram (see scale on the **right** side of the image) and in the values obtained for the shear velocity (chart **below** and on the **right**).

Another dynamic method, acoustic radiation force impulse (ARFI) elastography, has been applied to investigate mammary lesions in bitches. Studies regarding ARFI elastography demonstrated that malignant lesions were less deformable than benign ones. Where benign lesions tend to be deformable (that is, soft), malignant lesions have a tendency to be non-deformable (hard) and a cut-off value to differentiate benign lesions from malignant ones was proposed (SWE > 2.57 m/s), with 95% diagnostic accuracy, 98% AUC at 94.7% sensitivity and 97.2% specificity (Figure 3), demonstrating that the harder the lesion the higher the chances of malignancy [24,42].

Therefore, ARFI elastography has demonstrated promising results and could potentially be implemented in small animal practice. According to Feliciano et al., the increase in rigidity in mammary neoplasms is related to a stromal reaction induced by mammary carcinoma, increased levels of collagen and, possibly, the presence of fibrosis and microcalcifications detected in neoplasms [24,42]. Nevertheless, to this date, the availability of ultrasound machines equipped with specific elastography software is still mainly restricted to academic and research facilities due to the high cost of this equipment.

In 2018, Gasser et al. reported that no difference could be observed in quantitative measurements obtained by ARFI elastography to differentiate benign mammary lesions [31]. However, the authors report that patient movement, as well as the size of the electronic caliper available were hindering to the evaluation, thus presented important limitations of the study, potentially contributing to the results obtained.

When evaluating the types of carcinomas and grades, Feliciano et al. found that most of the masses were non-deformable and rigid, indicating malignancy, with high shear velocity (6.0 m/s) associated with low elasticity [32]. The authors also observed that special and complex carcinomas showed greater rigidity in the qualitative and quantitative evaluation, however they report the diagnostic technique is not sensitive enough for differentiation of these tumor types.

### 3.4. Contrast-Enhanced Ultrasound (CEUS)

**Figure 4:**
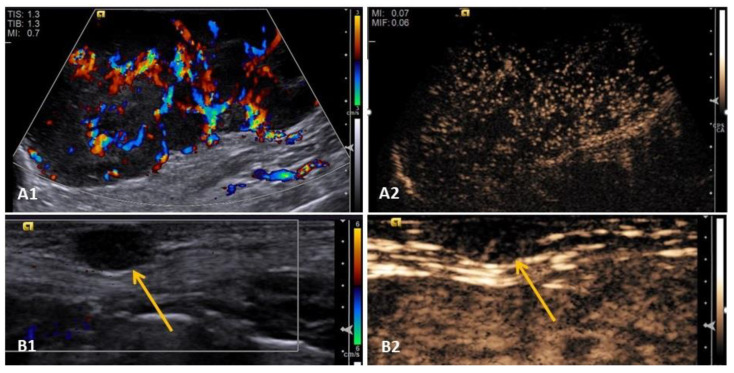
Contrast-enhanced ultrasound image of mammary neoplasms in bitches. (**A1,A2**) Grade III solid mammary carcinoma—note the difference in vascularity seen on color-coded Doppler (**A1**) and perfusion seen on CEUS (**A2**); Grade II mixed tumor mammary carcinoma (yellow arrows)—(**B1**) mammary nodule on color Doppler, with absence of neovascularization detected by this method and in (**B2**) presence of tissue perfusion detected by CEUS.

Contrast-enhanced ultrasound (CEUS) allows the evaluation of tissue perfusion in real time through the dynamic enhancement of the image by the presence or absence of enhancement by the contrast medium using parameters such as wash-in time (WI in seconds), wash-out time (WO in seconds), time to peak enhancement (TTP in seconds), as well as enhancement characteristics (enhancement compared to adjacent tissue), perfusion patterns (centripetal, centrifugal, or diffuse), localization (central, peripheral, or diffuse), internal homogeneity (homogenous or heterogenous), and perfusion type (discreet, moderate, or increased) [13,14,15,24] (Figure 4).

Studies regarding CEUS evaluation of mammary lesions in bitches demonstrated that perfusion parameters were not able to differentiate tumor types; however, those studies demonstrated that it was possible to identify tumors at a macro and microcapillary level, in addition to establishing reference values such as wash-out time, which, at <80.5 s, demonstrated a sensitivity of 80.2% and specificity of 16.7% for predicting malignancy [24]. The authors also found a higher peak of enhancement in benign lesions, which may be correlated with inflammatory processes or fibroadenomas, inadequate intratumoral angiogenesis, and insufficient capillary network involved in tissue development.

Despite not being as effective in detecting malignancy as elastography, contrast enhanced ultrasonography can be used in these tumors to identify vascularization, especially when this parameter is not verified by color-coded Doppler (Figure 4), demonstrating its potential to detect vascularization and tissue perfusion when Color-coded Doppler cannot [24],

In mammary carcinomas of bitches the contrast medium wash-in and peak enhancement times were significantly higher (*p* < 0.05) in simple and special types, and with cohort values lower than 7.5 s for wash-in times and lower than 13.5 s to peak enhancement were indicative of complex type. Contrast wash-in, peak enhancement, and wash-out times were significantly lower (*p* < 0.05) in the grade I, and with cohort values greater than 6.5 s for wash-in, 12.5 s for peak, and 64.5 s for wash-out times for grades II and III [32].

Another promising application of CEUS was demonstrated in 2019 by Abma et al., who reported the applicability of CEUS and power Doppler evaluation to assess the vascular response to anti-vascular therapy in bitches with mammary tumors, where it was possible to identify an important decrease in the enhancement type in the central region of the tumor with the progression of the treatment [43]. Therefore, CEUS evaluation could be a promising technique to investigate the progression of oncological treatments and further studies are warranted. However, similar to the availability of ultrasound units equipped with elastography, to this date, the availability of machines equipped with CEUS technology is also mainly restricted to academic and research institutions due to the cost of specific equipment and software programs required for CEUS evaluation and the cost of the contrast medium.

### 3.5. Regional Lymph Nodes

Considering the requirements for clinical staging of bitches with mammary tumors, the evaluation of lymph nodes is of extreme importance to establish adequate therapeutic management and prognosis [16]. The evaluation of sentinel lymph nodes in bitches with mammary tumors is essential to determine the prognosis of patients, as metastases of these neoplasms occur mainly in the inguinal and axillary locoregional lymph nodes and, when present, the prognosis is unfavorable [36]. The adequate evaluation of these structures is important to determine the staging of neoplasms, choose the best treatment approach, carry out adequate planning for removal of surgical margins and define the prognosis. Definitive diagnosis of regional lymph node metastasis is performed on histopathology by surgical excision, a procedure with high complications risk, due to proximity of large vessels and nervous plexus to the axillary lymph node [16]. The applicability of the ultrasonographic evaluation of these sentinel lymph nodes may potentially guide the practitioner as to the need of their surgical removal, thus reducing surgical risks and incidence of complications.

On ultrasound evaluation, common features to assess regional lymph nodes are the short/long axis ratio (S/L), echogenicity, blood flow distribution, and hemodynamic indices where benign lymph nodes are considered to be homogenous hypoechoic, present a S/L < 0.5, hilar blood flow distribution on color-coded Doppler assessment, and a resistive index (RI) < 0.65 [44]. However, divergent findings have been reported regarding the diagnostic accuracy of B-mode and color-coded Doppler findings of axillary and inguinal lymph nodes of bitches with mammary tumors [45,46].

In a study carried out by Silva et al., regional lymph nodes were studied by ultrasound examinations of 100 bitches affected by mammary tumors (100 inguinal and 96 axillary), detecting healthy, reactive, and metastatic tissues by means of histopathology of the locoregional lymph nodes. According to the authors, ultrasonographic evaluation of the lymph nodes was performed without difficulties, demonstrating the feasibility of the method in the evaluation of these structures in small animal practice [36].

According to Muramoto et al. it is not possible to establish reference standards for measurements of locoregional, due to the great variability of findings [47]. B-mode findings as the presence of necrotic, liquefaction, or hemorrhagic areas, normal areas and with tumor alteration tissues, micro calcifications and gross calcifications, results in visualization of heterogeneous echotexture in all tissues evaluated, making it difficult to use this method in detection of metastasis in lymph nodes [45]. Stan et al. [46] reports that S/L can be an accurate measurement to distinguish between benign and malignant tumors in bitches with mammary tumors, where S/L > 0.55 presents a sensitivity of 83.3% and specificity of 78.6% to detect metastatic lymph nodes.

In relation to Doppler and its ineffectiveness in differentiating metastasis in sentinel lymph nodes, according to Silva et al., variations in the vascularization of neoplastic and non-neoplastic tissues due to inflammatory factors, promotes detectable fluxes in normal and reactive lymph nodes and even less evident vascularization in ischemic foci derivate from metastatic lymph nodes necrosis, making it impossible to use Doppler for diagnostic purposes in lymph nodes [45]. Conversely, Stan et al. [46] reports that hemodynamic indices obtained by Pulsed-wave Doppler can help differentiate benign from malignant processes in the affected lymph node, where a cutoff value for resistive index >0.56 and pulsatility index >1.02 were suggestive of metastatic infiltration.

Another important application of ARFI elastography in bitches with mammary tumors is the evaluation of locoregional lymph nodes. In a study, Silva et al., the authors found a difference in stiffness between metastatic, reactive, and normal lymphoid tissues; with the metastatic sentinel lymph nodes more rigid than the reactive and normal ones, with the shear wave velocity enabled metastasis identification in inguinal (sensitivity 95% specificity 87%) and axillary lymph nodes (sensitivity 100% specificity 94%), and with cohort value >2.5 m/s for shear velocity in metastasis identification [45]. Abnormal cell proliferation, microcalcifications, and deposition of abnormal tissues in the stroma of sentinel lymph nodes are related to the increase in stiffness detected by ARFI elastography in sentinel lymph nodes [36,39].

Using strain elastography, Stan et al. [46] reported that the elasticity score is the most reliable feature to differentiate between benign and malignant tumors, where a subjective elasticity score is given to the tissue and, the harder the lymph node, the higher the chances of metastatic infiltration. Additionally, these same authors investigated the applicability of CEUS evaluation of regional lymph nodes, where it was described that the shorter and more marked wash-out time, the higher the chances of metastatic infiltration.

## 4. Conclusions

The prevalence of mammary tumors in bitches is a concerning matter in small animal practice, therefore, early investigation and diagnosis is crucial for adequate patient care. Although definitive diagnosis is obtained by histopathological evaluation, mammary ultrasonography has been demonstrated as a great tool for the investigation of mammary lesions and has presented great results in a non-invasive and accessible way.

There are divergences regarding B mode ultrasonography features of mammary lesions that could discriminate between benign and malignant mammary lesions. Color-coded Doppler evaluation provides great information regarding the presence or absence of blood flow within a mammary lesion and when combined with pulsed-wave Doppler, it can provide hemodynamic information regarding the lesion, which can be helpful in addressing the most likely diagnosis. Elastography demonstrates great accuracy to evaluate mammary masses, as well as lymph nodes and presents great potential for use in small animal practice. CEUS assessment does not provide a good discrimination between benign and malignant lesions, but it does provide information regarding tissue perfusion.

The different ultrasonographic methods (B mode, Doppler, elastography, and CEUS), when applied together and carefully interpreted along with other clinical, physical, and laboratory findings can be helpful for the practitioner to establish an adequate therapeutic approach. However, it is important to remember that the gold standard is histological evaluation, and that definitive diagnosis cannot be obtained by means of ultrasonography. Additionally, it is prudent to consider that, unfortunately, ultrasonographic assessment of the mammary glands is only implemented when mammary lesions are visible and palpable; therefore, to this date, it is not an early diagnostic technique. Furthermore, ultrasonography demonstrates a promising future and new studies are still warranted to further explore the applicability of these techniques to investigate the therapeutic progression of bitches with mammary neoplasms.

## Figures and Tables

**Table 1:** Modified BI-RADS™ Ultrasound Lexicon for the Evaluation of Canine Mammary Tumors.

Findings	Term	
Nodules/masses	1. Shape	a. Oval b. Round c. Irregular
	2. Orientation	a. Parallel b. Not parallel
	3. Margins	a. Circumscribed b. Not circumscribed I. Indistinct II. Angular III. Microlobulated IV. Spiculated
	4. Echo pattern	a. Anechoic b. Hyperechoic c. Complex cystic and solid d. Hypoechoic e. Isoechoic f. Heterogenous
	5. Posterior features	a. No posterior feature b. Enhancement c. Shadowing d. Combined pattern
Calcification	1. Calcification in a mass 2. Calcification outside of a mass 3. Intraductal calcification	
Associated features	1. Architectural distortion	
	2. Duct changes	
	3. Skin changes	a. Skin thickening b. Skin retraction
	4. Edema	
	5. Vascularity	a. Absent b. Internal vascularity

Adapted from Mohammed et al., 2011; ACR BI-RADS™ 2013; and Oliveira et al., 2018 [34,36,37].

**Table 2:** Modified BI-RADS™ categories and their respective sonographic findings.

Modified BI-RADS Category	Sonographic Findings
Category 1	Normal examination
Category 2	Intramammary lymph nodes, benign calcifications, fat nodules
Category 3	Solid hypoechoic circumscribed oval structures with parallel orientation, simple cysts
Category 4	**4a:** Partially circumscribed mass, complex cysts **4b:** Not circumscribed oval or rounded solid masses, grouped calcifications **4c:** Irregular shape not circumscribed masses, new group of calcifications
Category 5	Irregular shape, not circumscribed, non-parallel orientation, hyperechoic halo, posterior acoustic shadowing, alterations in adjacent tissues
Category 6	Proven malignancy by biopsy

Adapted from Mohammed et al., 2011; ACR BI-RADS™ 2013; and Oliveira et al., 2018 [34,36,37].

## Data Availability

The data presented in this study are available on request from the corresponding author for scientific purposes.
